# Health Resorts

**Published:** 1902-08-02

**Authors:** 


### HEALTH RESORTS.

### BOURNEMOUTH.

Among the various watering-places on the south coast there are few which offer attractions to so large a range of clients as does Bournemouth. In the early days of its growth as a health resort Bournemouth depended for its popularity upon its warm and sheltered position. Built round the opening of a valley—the bourne mouth—by which the cliffs to the east and the west of it are cut in two, and nestling in the midst of numerous trees, the houses were well protected from the prevailing and more violent winds, and yet were exposed to a sufficient current of moving air. Thus Bournemouth in the early days became synonymous in the minds of many with rest, protection, quietness, and warmth, the sort of climate which suited bronchitis and kidney disease, and many cases of advanced consumption in which alleviation rather than cure was the object aimed at. And Bournemouth still gives all this to those who want it. But in its growth the town soon spread beyond the comparatively narrow limits of the early settlement. It grew along the cliffs and out into the open country, and now covers many elevated districts which are open to all the winds of heaven; and so it happened that almost coincidently with the great change in medical opinion which has led to the demand for air, and still more air, in the treatment of many morbid conditions, Bournemouth has had the good luck to have grown out from its narrow home on to the breezy cliffs and uplands. To this no doubt is largely due the extraordinary prosperity which has befallen the town. It has long ceased to be the almost exclusively invalid resort that it used to be, it has grown to be a great town of over 60,000 inhabitants, with all the attributes of active municipal life, has become a great pleasure resort to which thousands of pleasure seekers flock year after year, and in addition to its invalid season in the winter has developed a great bathing season in the summer. All of which is good for the invalids. Sick folk depress each other when they are too closely packed. Bournemouth stands on an extensive plateau of sandy soil, about 100 feet above the sea level, which is divided by various chines or valleys, the principal one being that near the mouth of which the centre of the town is situated. It is a handsome, busy, and extremely beautiful town. The eastern cliff is well wooded, the western is more open, as also are the northern portions. Within half a mile of the centre one may be buried in the quietest seclusion, but the town itself is full of life, a pier, beautiful gardens, a winter garden, admirable music, theatres, and many kinds of amusement are constantly going on. The churches are numerous, the services well conducted. The hotels are good, while carriages, cabs, omnibuses, char-a-bancs and motor-cars provide the necessary means of communication. Doctors and specialists of many kinds abound, and the various forms of "cure" used in chest diseases are more particularly in evidence. With all this we have a town which is not only a "health resort," but is also itself extraordinarily healthy, a combination not always met with. According to the last annual report of the medical officer of health the death-rate for the year 1901 was 11·5 per 1,000, while the zymotic death-rate was only 0·3 per 1,000. The town is provided with an isolation hospital and other modern sanitary arrangements. In regard to the cases which may properly be sent there one must remember that it is now a summer as well as a winter resort, and that while in winter all sorts of cases which require a protective climate—bronchitis, kidney disease, arterial degeneration, certain cases of phthisis, old people and children recovering from illness—may be sent there; in summer it is a great pleasure resort and place for convalescence. All the year round, however, open-air cures are carried on.

### LLANDRINDOD WELLS.

The little town of Llandrindod Wells, a watering-place which has lately come into considerable note, is situated in a hilly district in the County of Radnor, in South Wales. In a sense the place is old, the churches around being ancient, and the waters themselves having been well known for their medicinal properties for several hundreds of years. But the town is modern, the greater part of it having been built within the past twenty years. In 1891 the resident population numbered 784, while at the census last year they numbered 1,828. To these in the height of the season must be added at least 5,000 visitors. During the past five years many improvements have been made, including the introduction of electric lighting and the construction of concrete footways, new sewers, and water supply. The slope of the land makes it possible to deal easily and effectually with the sewage, which before its discharge is treated on the septic tank system. Geologically, Llandrindod Wells stands upon the Llandeilo flags of the Silurian system at a point in the neighbourhood of which igneous rocks have forced their way through the overlying strata. Around these igneous intrusions the fissures in the shale contain veins of iron pyrites (sulphide of iron), and it is no doubt by the decomposition of these that some of the important constituents of the waters are derived.

The waters are all strongly saline, being highly charged with common salt, and with the exception of the strong sulphur water they all contain a considerable amount of chloride calcium. They also all contain magnesium salts of one kind or another, and varying proportions of the many other mineral ingredients always found in similar circumstances. Therapeutically they may be divided into three groups:—(1) the iron waters, (2), the saline waters, and (3) the sulphur waters. Both the iron and the saline waters contain a fair quantity of magnesium salts. The sulphur waters, on the other hand, contain less of the saline elements, but are charged with sulphuretted hydrogen gas. Evidently such waters possess a considerable range of utility. Dr. Floyd, the medical officer of health, says that the cases which derive most benefit are those of gout, rheumatism, chronic skin diseases, tuberculosis, diabetes, and disorders due to errors in diet. Perhaps we may add that the term "errors in diet" must be taken to include errors taking the direction of alcoholic excess, for the place has a certain repute, not only in the treatment of early cirrhosis of the liver and other ailments which are generally associated with alcoholism, but also in diminishing the drink craving which is in some such cases so difficult to overcome. Perhaps one may doubt how far Llandrindod Wells, or indeed any other wells, are likely to prove effectual in the treatment of true dipsomania; but there are a great number of patients who become alcoholic merely as a result of "not feeling well," which in turn arises from a variety of disorder of the digestive organs which are capable of distinct and rapid relief by the waters of Llandrindod Wells, and when the dyspeptic miseries are relieved, the "lowness" and the "craving" vanish likewise. Hence, probably, the reputation of this place in such cases. It is usual to drink the waters early in the morning, fasting, and to take brisk exercise between the successive glasses; and, according to local opinion, the waters are regarded as not agreeing unless they act in a laxative manner. As to this we would emphasise one point, namely, that where, as is the case here, there are several springs containing different ingredients, and these in very different proportion, it is highly important that local medical advice should be obtained as to the manner and quantity in which they should be taken. No amateur attempts should be made to run a course on one's own account. Llandrindod Wells stands 700 feet above the sea level, and at some little distance away there are other higher hills of far greater elevation; the place is easily accessible by railway, and is well provided with all invalid requirements, while the country around is sufficiently interesting to provide amusement for the large number of healthy visitors who merely wish for a holiday in the pure air of an elevated region.

